# Gut Microbiota Interventions With *Clostridium butyricum* and Norfloxacin Modulate Immune Response in Experimental Autoimmune Encephalomyelitis Mice

**DOI:** 10.3389/fimmu.2019.01662

**Published:** 2019-07-23

**Authors:** Hao Chen, Xiaomeng Ma, Yingying Liu, Lili Ma, Zhaoyu Chen, Xiuli Lin, Lei Si, Xueying Ma, Xiaohong Chen

**Affiliations:** Department of Neurology, The Third Affiliated Hospital of Sun Yat-sen University, Guangzhou, China

**Keywords:** *Clostridium butyricum*, norfloxacin, Th17/Treg, gut microbiota, EAE, multiple sclerosis

## Abstract

Gut microbiota has been proposed as an important environmental factor which can intervene and modulate central nervous system autoimmunity. Here, we altered the composition of gut flora with *Clostridium butyricum* and norfloxacin in experimental autoimmune encephalomyelitis (EAE), an animal model of multiple sclerosis. We found that appropriate *C. butyricum* (5.0 × 10^6^ CFU/mL intragastrically daily, staring at weaning period of age) and norfloxacin (5 mg/kg intragastrically daily, 1 week prior to EAE induction) treatment could both ameliorate EAE although there are obvious differences in gut microbiota composition between these two interventions. *C. butyricum* increased while norfloxacin decreased the abundance and diversity of the gut microbiota in EAE mice, and both of the treatments decreased *firmicutes*/*bacteroidetes* ratio. In the genus level, *C. butyricum* treatment increased the abundance of *Prevotella* while *Akkermansia* and *Allobaculum* increased in norfloxacin treatment. Moreover, both interventions reduced *Desulfovibroneceae* and *Ruminococcus* species. Although there was discrepancy in the gut microbiota composition with the two interventions, *C. butyricum* and norfloxacin treatment both reduced Th17 response and increased Treg response in the gastrointestinal tract and extra-gastrointestinal organ systems in EAE mice. And the reduced activity of p38 mitogen-activated kinase and c-Jun N-terminal kinase signaling in spinal cord could be observed in the two interventions. The results suggested that manipulation of gut microbiota interventions should take factors such as timing, duration, and dosage into consideration. The discrepancy in the gut microbiota composition and the similar protective T cells response of *C. butyricum* and norfloxacin implies that achieving intestinal microecology balance by promoting and/or inhibiting the gut microbiota contribute to the well-being of immune response in EAE mice.

## Introduction

Multiple sclerosis (MS) is a chronic autoimmune inflammatory demyelinating disease of the central nervous system (CNS) and its incidence continues to increase worldwide (1). The pathogenesis of MS is complex and therapeutic effects are not satisfying. Besides genetic factors, environmental factors also play an important role in pathogenesis of MS. Among them, gut microbiota can make a major contribution to the disease both in susceptibility and protection (2–4). Thus, the discovery of MS-related pathogenic and/or protective gut microbiota organisms or their products would provide novel opportunities for diagnosis and therapy of the disease. The latter would mainly rely on appropriate probiotic and/or antibiotic treatments, dietary modification, and fecal microbial transplantation.

Probiotics are live bacteria that can exert beneficial effects on the host when administered in appropriate amounts. *Clostridium butyricum* (*C. butyricum*) is a butyric acid-producing bacterium which has been widely used for improving gastrointestinal function as probiotics. It has been demonstrated to ameliorate experimental colitis and asthmatic in mice by reversing the imbalance of Th1/Th2 through IL-10 dependent mechanism (5, 6). *C. butyricum* also can protect against autoimmune diabetes in mice by modulating Treg/Th17 differentiation and generate a less pro-inflammatory immunological microenvironment in the gut (7). Moreover, it has been used for treatment of human gastrointestinal disease in clinical practice (8, 9). Rare reports was investigated the effects of *C. butyricum* on experimental autoimmune encephalomyelitis (EAE), a classical animal model of MS.

Antibiotics can easily affect the components of the gut microbiota and alleviate EAE (10, 11). The study of antibiotics on EAE did not focus on gut microbiota, but on other EAE pathogenesis-related components (12). Norfloxacin is a fluoroquinolone antibiotic which has pleiotropic effects beyond its bactericidal effect (13–15). It exerts immunomodulatory effects via maintaining low endotoxin levels and stimulating the production of IL-10 in experimental cirrhosis mice (13, 14). Moreover, norfloxacin inhibits lipopolysaccharide-induced pro-inflammatory cytokine production such as tumor necrosis factor-α (TNF-α) and interleukin-1β (IL-1β), then modifies inflammatory responses (15). However, gut microbiota intervention with norfloxacin on EAE has never been examined.

In the present study, *C. butyricum* and norfloxacin were selected as gut microbiota interventions on EAE. We found that appropriate *C. butyricum* and norfloxacin can ameliorate EAE although there are obviously different changes in gut microbiota composition between the two interventions. The benefit of these two interventions is associated with reduced Th17 response and increased Treg response in the gastrointestinal (GI) tract and extra-GI organ systems in EAE mice. And the reduced activity of p38 p38 mitogen-activated kinase (MARK) and c-Jun N-terminal kinase (JNK) signaling may contribute to the molecular mechanisms of these benefit effects.

## Materials and Methods

### Mice and Reagents

C57BL/6J WT female mice were purchased from Guangdong Medical Laboratory Animal Center (Guangzhou, China). All mice were maintained under specific pathogen free conditions at South China Agricultural University (Guangzhou, China). The mice were allowed to acclimatize to the laboratory for 1 week prior to beginning of the study. All experiments were performed using female mice in accordance with guidelines for animal care, created by according to the National Institutes of Health Guide for Care and Use of Laboratory Animals and approved by the Bioethics Committee of South China Agricultural University (Approval ID: 2018-D006). *C. butyricum* powder (GDBIO1501, GDBIO-TECH Biotechnology) was stored at −20°C. Norfloxacin were purchased from Ji Lin SUNFUNG MEDCINE Co., Ltd, and MOG35-55 peptide (MEVGWYRSPFSRVVHLYRNGK) was synthesized by CL. BioScientific CO., LTD (Xi'an, China). Amino acid sequences were confirmed by aminoacid analysis and mass spectroscopy. The purity of the peptide was >95%. *Mycobacterium tuberculosis* H37RA was purchased from Difco (Detroit, MI). Pertussis toxin (PTX) was purchased from Alexis Corp (San Diego, CA). FITC- or PC-5.5-conjugated anti-mouse CD4, PC-7A-conjugated anti-mouse IFN-γ (interferon-gamma), PE-conjugated anti-mouse IL-17A, PE-conjugated anti-mouse Foxp3 were purchased from eBioscience (San Diego, CA, USA). P38 MAPK, p-p38 MAPK, extracellular signal-regulated kinase (ERK) 1/2, p-ERK 1/2, JNK, p-SAPK/JNK were purchased from Cell Signaling Technology (USA).

### Dose-Finding Experiments and Treatment of Mice

Mice were randomly assigned to four groups: control mice, PBS-treated EAE mice, *C. butyricum*-treated EAE mice, and norfloxacin-treated EAE mice. The concentrations of *C. butyricum* and norfloxacin were chosen on the basis of previous *in vivo* data and our preliminary dose-finding experiment. Because 3–4 weeks (weaning period) of age, is an ideal time for probiotic intervention (7, 16), 5.0 × 10^5^, 5.0 × 10^6^, and 5.0 × 10^7^ CFU/mL concentrations of *C. butyricum* in PBS (200 μL) was intragastrical administration 3 weeks before EAE induction, and 5.0 × 10^6^ CFU/mL concentration of *C. butyricum* was selected as the optimal dosage. One week prior to EAE induction, mice received norfloxacin in PBS 5 mg/kg intragastrically daily (14, 17).

### Induction and Assessment of EAE

EAE was induced by the procedure which had been described previously (18, 19). Briefly, 6–8 week female mice received a subcutaneous injection in the flanks with 300 μg of MOG35-55 peptide per animal emulsified in CFA containing 500 ug of *Mycobacterium tuberculosis* H37RA. Immediately thereafter, and again 48 h later, the mice received an intraperitoneal (i.p.) injection of 300 ng of PTX in 100 μL of phosphate buffered saline (PBS). An additional injection of MOG35-55 peptide in CFA was delivered 7 days later. The animals were examined daily for disability. Clinical assessment of EAE scores was performed daily on a scale of 0–5 as instructed (19).

### Histology Evaluation

After 21 days post immunization, different treated mice (*n* = 6) were fixed by heart perfusion with 4% (w/v) paraformaldehyde, and the lumbosacral spinal cords were obtained and embedded in paraffin. Paraffin sections were stained with hematoxylin and eosin and solochrome cyanin impregnation for evaluating inflammatory infiltration and demyelination, respectively. The scale evaluated for inflammation was as follows (20): 0, no inflammatory cells; 1, a few scattered inflammatory cells; 2, organization of inflammatory infiltrates around blood vessels; 3, extensive perivascular cuffing with extension into adjacent parenchyma, or parenchymal infiltration without obvious cuffing. Demyelination in the spinal cord was scored as previously described (21, 22): 1, traces of subpial demyelination; 2, marked subpial and perivascular demyelination; 3, confluent perivascular or subpial demyelination; 4, massive perivascular and subpial demyelination involving one half of the spinal cord with presence of cellular infiltrates into CNS parenchyma; 5, extensive perivascular and subpial demyelination involving the whole cord section with presence of cellular infiltrates into CNS parenchyma.

### 16S rRNA Gene Sequencing

Fresh extruded stools were collected before EAE treatment and immediately positioned in carbon dioxide ice for the gut microbial analysis. Feces DNA was extracted using QuickGene DNA tissue kit from Kurabo Company (Neyagawa, Japan) and sent for PCR amplification and sequencing of the V3 and V4 region of bacterial 16S rRNA genes using the Illumina MiSeq technology at BGI Co. (Shenzhen, China).

For alpha diversity analysis, we rarified the OTU to several metrics, including curves of OTU rank, rarefaction and Shannon, and calculated indexes of Shannon, Chao1, Simpson, and ACE. For beta diversity analysis, principal component analysis (PCA) was performed using QIIME (23). The LDA effect size (LEfSe) analysis was performed for the quantitative analysis of biomarkers among each group (24). Briefly, LEfSe analysis, LDA threshold of >4, used the non-parametric factorial Kruskal-Wallis (KW) sum-rank test and then used the (unpaired) Wilcoxon rank-sum test to identify the most differently abundant taxa.

### Intracellular Cytokine and Intra-Nuclear Foxp3 Staining

For intracellular cytokine staining, lymphocytes isolated from designated organs 21 days after immunization were stimulated, fixed and permeabilized as instructed (25), followed by fluorescent-conjugated intracellular cytokine antibody staining. Intra-nuclear fork-head box p3 (Foxp3) was stained using the Foxp3 Staining Buffer Set (eBioscience, San Diego, CA, USA).

### Western Blot

To investigate the protein expression of p38 MAPK, p-p38 MAPK, ERK1/2, p-ERK1/2, JNK, p-JNK in the spinal cord of control mice, PBS-treated EAE mice, *C. butyricum*-treated EAE mice, and norfloxacin-treated EAE mice, we performed Western blot analysis. Samples of the spinal cord from differently treated mice were loaded on 10% gradient sodium dodecyl sulfate-polyacrylamide gels (20 mg protein per lane). Proteins were transferred onto PVDF membrane (Bio-Rad). The membranes were blocked by 5% non-fat milk. Afterward, the membranes were incubated with p38 MAPK (1:1,000), p-p38 MAPK (1:1,000), ERK1/2 (1:1,000), p-ERK1/2 (1:1,000), JNK (1:1,000), p-JNK (1:1,000) overnight, respectively. After three times, washes with TBST buffer, the membrane was incubated with anti-mouse-HRP and goat anti-rabbit-HRP for 30 min, respectively. The experiment was repeated in triplicate and β-actin was used as internal control.

### Statistical Analysis

Data were expressed as mean ± s.d., except for the clinical EAE score, which was expressed as mean ± s.e.m. Differences between two groups were analyzed by a two-tailed Student's *t*-test. ANOVA was used to compare difference of data from more than two groups, and the non-parametric data. Statistically significant data are indicated by asterisks (^*^P < 0.05, ^**^P < 0.01, ^***^P < 0.001, and ^****^P < 0.0001). Experiments were repeated three time.

## Results

### Gut Microbiota Interventions With *C. butyricum* and Norfloxacin Ameliorated EAE

**Figure 1:**
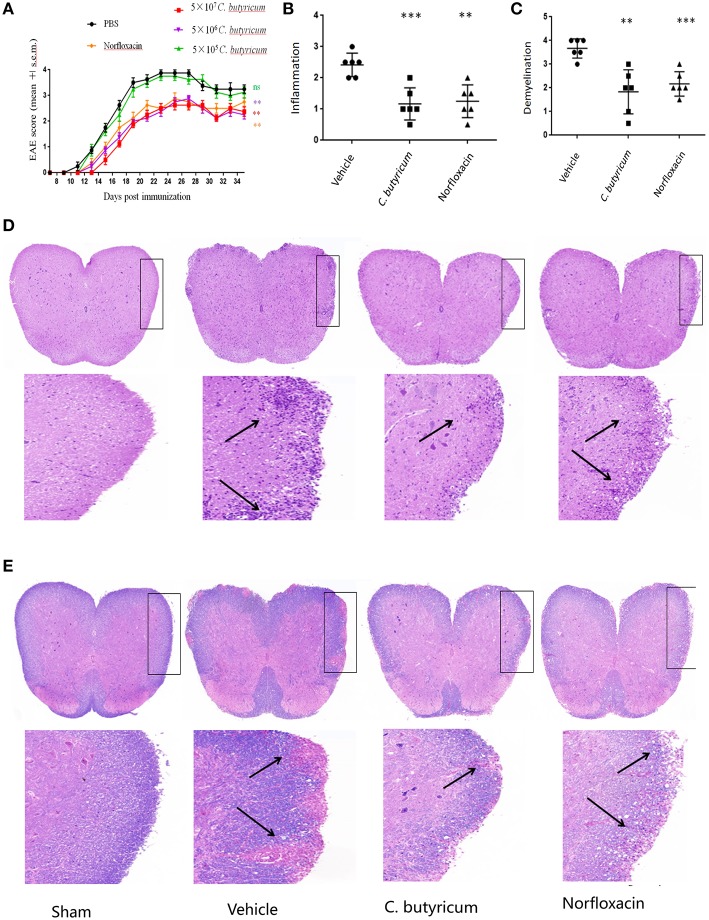
*C.butyricum* and norfloxacin treatment both ameliorated clinical severity and neuropathology of EAE mice. **(A)** Clinical score was assessed daily and shown (*n* = 8). **(B–E)** At 21 days post immunization, lumbosacral spinal cords were isolated and performed H&E (*n* = 6) **(D)** or Solochrome cyanin impregnation **(E)** staining for assessment of histopathology. Representative sections **(D,E)** and statistical analysis **(B,C)** data are shown. The infiltration of lymphocytes and demyelination are highlighted by arrow. Experiments were repeated three times with similar results. Statistically significant data are indicated by asterisks (^**^P < 0.01 and ^***^P < 0.001).

In an attempt to study the effects of the gut intervention on EAE, *C.butyricum* and norfloxacin treatment were used. We found that 5.0 × 10^5^ CFU/mL *C. butyricum* treatment could not produce protective effects on EAE mice. Both 5.0 × 10^6^ and 5.0 × 10^7^ CFU/mL *C. butyricum* could suppress the severity of EAE while there were no significant differences between these two groups. Therefore, 5.0 × 10^6^ CFU/mL was selected as the optimal dose in the following experiments. Results showed that both treatments significantly ameliorated the disease severity, as evidenced by reduced disease score (Figure 1A). As for neuropathology, both treatments could decrease lymphocyte infiltration and plaques of demyelination in lumber spinal cord (Figures 1B–E). These results indicated that gut microbiota interventions with *C. butyricum* and norfloxacin both could ameliorate clinical severity and neuropathology of EAE mice. And we did not find any obvious adverse effects of *C. butyricum* or norfloxacin at the chosen doses.

### Gut Microbiota Interventions With *C. butyricum* and Norfloxacin Reconstituted the Composition of Intestinal Flora in EAE Mice

**Figure 2:**
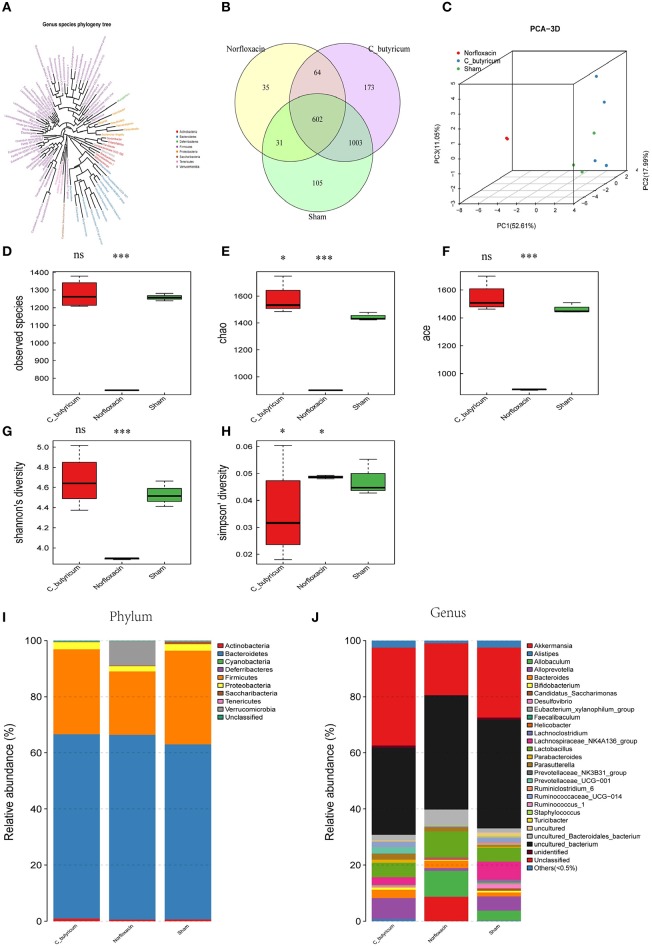
Responses of the diversity, richness, and structure of the gut microbiota to sham, *C. butyricum*, and norfloxacin treatment in EAE mice. **(A)** Genus species phylogeny tree of all three groups. **(B)** Venn diagram of each group and the number of differences between species are shown. **(C)** Unweighted Unifrac principal coordinates analysis plots 3D of each sample and the distance of each group are shown. Each sample was collected from three mice (*n* = 3). **(D–H)** shows the Observed species index, Shannon index, Chao index, Simpson index, and ACE index of each group. **(I–J)** Relative abundances of the gut microbiota at phylum level and genus level. Statistically significant data are indicated by asterisks (^*^P < 0.05 and ^***^P < 0.001). ns, not significant.

In order to investigate the gut microbial profile under *C. butyricum* and norfloxacin treatment, we analyzed the fecal bacteria from EAE mice by sequencing the bacterial 16s rRNA V3+V4 region. Based on 97% similarity level, all of the effective reads were clustered into operational taxonomic units (OTUs). The intestinal microbiota structural changes were then analyzed using unsupervised multivariate statistical methods PCA. Genus species phylogeny tree revealed the relationship between intestinal flora compositions in mice (Figure 2A). As shown in Figures 2B–J, three groups presented a distinct clustering of microbiota composition and norfloxacin decreased the number of OTUs while *C. butyricum* increased the numbers of OTUs. The observed species (Figure 2D), as well as the indexes of Chao1 (Figure 2E), ACE (Figure 2F), Shannon (Figure 2G), and Simpson (Figure 2H) were calculated. Consistent with the number of OTUs, norfloxacin treatment significantly reduced both the abundance and diversity while *C. butyricum* treatment increased the abundance and diversity of the intestinal microbiota.

**Figure 3:**
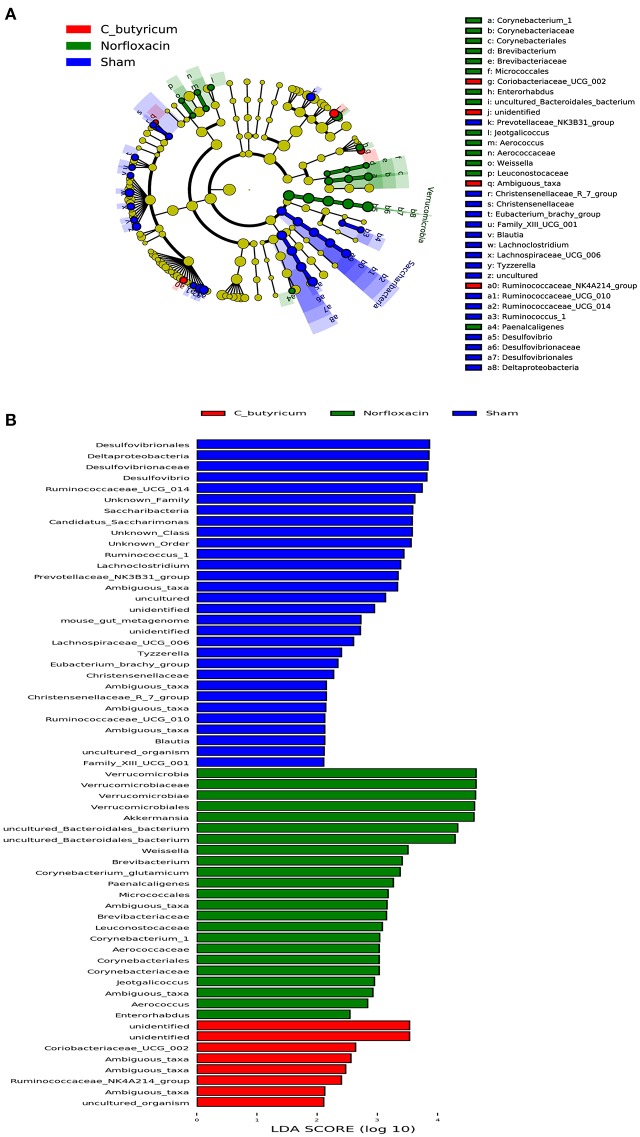
Identification of differential microbes in response to *C.butyricum* and norfloxacin treatment in mice based on the linear discriminant analysis (LDA) and effect size (LEfSe) pipeline. **(A)** Cladogram using LEfSe method indicated the phylogenetic distribution of gut microbiota associated with mice in three groups. **(B)** LDA scores showed the significant bacterial differences in three groups.

As shown in Figures 2I,J, the phylum level and genus level analysis demonstrated that both *C. butyricum* and norfloxacin administration significantly increased the relative abundance of *Bacteroidetes* and decreased the relative abundance of *Firmicutes*. Furthermore, norfloxacin notably increased the abundance of *Verrucomicrobia*. LEfSe and LDA score were employed to identify the differential distribution of microbiota among three groups (Figure 3). We found that *C. butyricum* induced a trend of a large increase in *Prevotella* while norfloxacin administration induced a trend of a large increase in *Akkermansia* and *Allobaculum*. Both *C. butyricum* and norfloxacin treatment can reduce the abundance of *Desulfovibroneceae* and *Ruminococcus*. Collectively, these results revealed the reconstitution of the gut microbiota composition in EAE mice when treated by *C. butyricum* or norfloxacin.

### Gut Microbiota Interventions With *C. butyricum* and Norfloxacin Suppressed Th17 Cells Response and Increased Treg Response in EAE Mice

**Figure 4:**
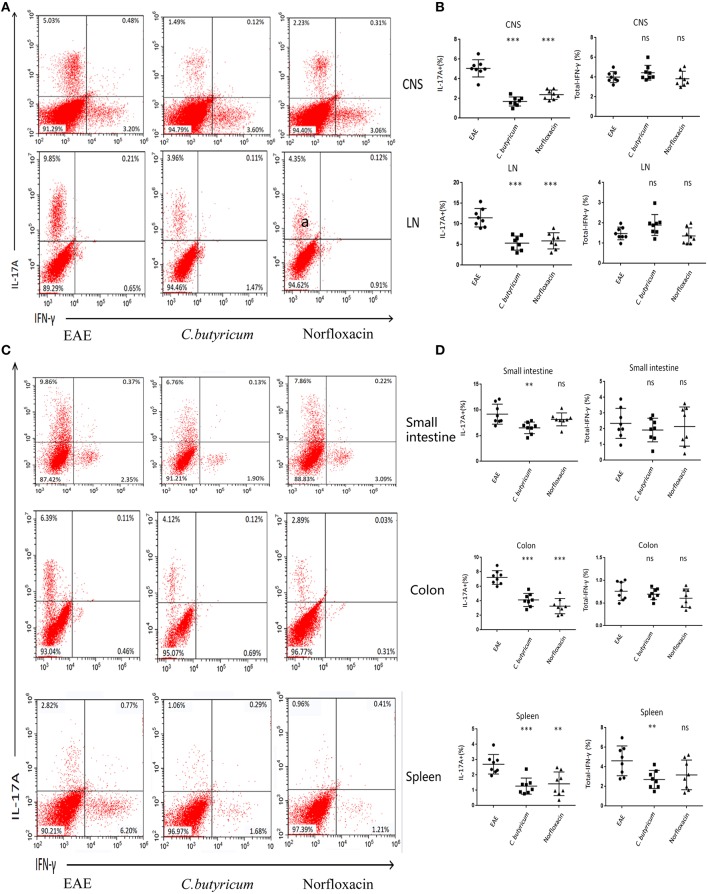
Gut microbiota intervention diminish Th17 responses in EAE. Lymphocytes from CNS, LN, small intestine, colon, and spleen were isolated 21 days post immunization and used for assessment of different CD4 T cell subsets. **(A)** Representative staining of different CD4 T cell subsets in CNS and LN, gated on TCRβ^+^ CD4^+^. **(B)** Statistical analysis of the percentages of IL-17 and IFN-γ in **(A)**. Representative staining **(D)** and statistical analysis **(C)** of IL-17 and IFN-γ in CD4 T cells isolated from small intestine, colon, and spleen of mice, gated on TCRβ^+^ CD4^+^. Statistically significant data are indicated by asterisks (^**^P < 0.01 and ^***^P < 0.001). ns, not significant.

**Figure 5:**
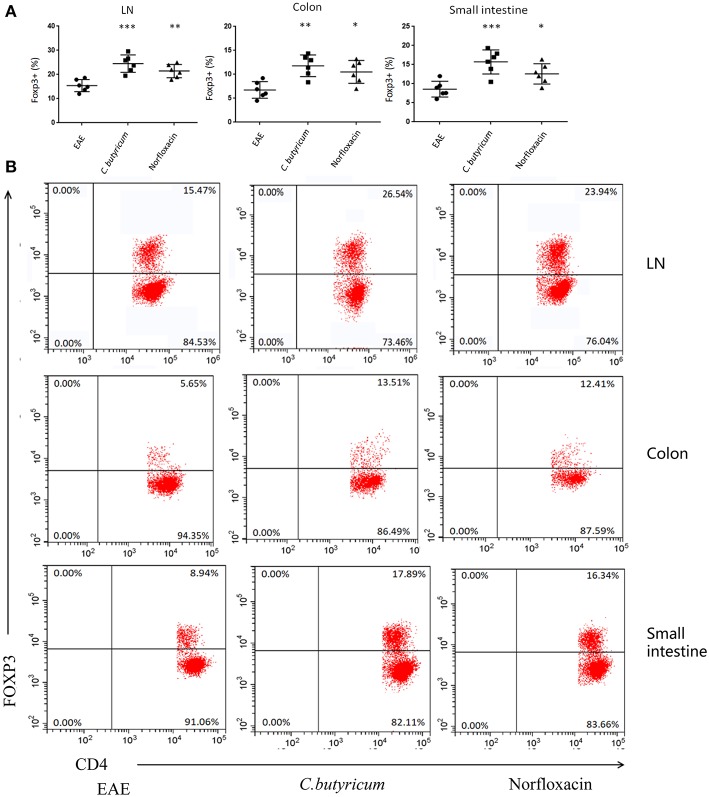
Gut microbiota intervention expanded the Treg cells in EAE. **(B)** Representative staining of different CD4 T cell subsets in inguinal LN, colon and small intestine, gated on TCRβ^+^ CD4^+^. Statistical analysis of data in **(A)**. Statistically significant data are indicated by asterisks (^*^P < 0.05, ^**^P < 0.01, and ^***^P < 0.001).

EAE is caused by aberrant T-cell responses to myelin self-peptides (26). And alterations of gut microbiota are involved in the changed T cells response in autoimmune diseases in the gastrointestinal (GI) tract and extra-GI organ systems (27). We further detected the levels of Th17 (CD4^+^ IL-17A^+^) and Th1 (CD4^+^ IFN-γ^+^) in the CNS, inguinal lymph nodes (LN), spleen, small intestine, and colon (Figure 4). And CD4^+^ Foxp3^+^ Treg cells in the LN, spleen, colon and small intestine were also analyzed in EAE mice (Figure 5). Results showed that *C. butyricum* reduced Th17 cells in all above-mentioned regions while norfloxacin reduced Th17 cells in CNS, LN, colon, and spleen except small intestine (Figures 4A–D). When it comes to Th1 cells, only the proportion of IFN-γ producing CD4^+^ T cells in the spleen from the *C. butyricum* group was reduced (Figures 4A–D). And no significant differences were noted in the percentages of Th1 cells in norfloxacin treatment in EAE mice (Figures 4A–D). We also found no statistical differences in IL-17A^+^ IFN-γ^+^ positive CD4 T cells in the treated groups (data not shown). Moreover, Treg cells decreased in the above regions while treatment with *C. butyricum* and norfloxacin promoted the Treg differentiation in EAE mice (Figures 5A,B).

### Gut Microbiota Interventions With *C. butyricum* and Norfloxacin Suppressed the MAPK Pathway in EAE Mice

**Figure 6:**
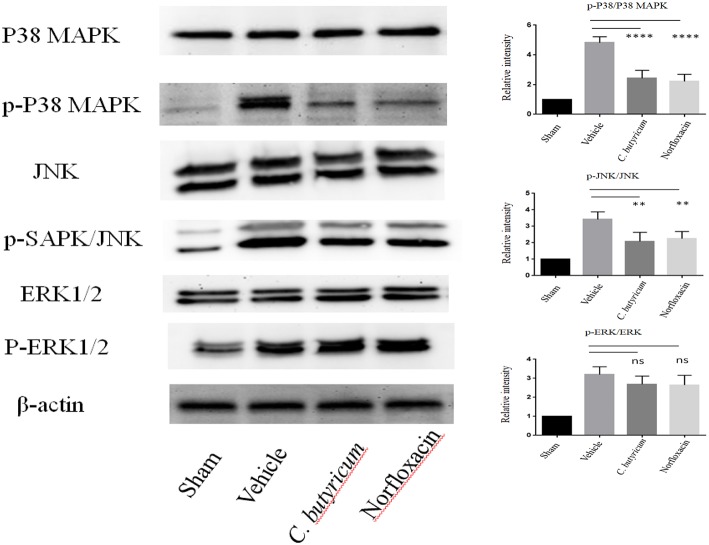
Gut microbiota intervention suppressed the phosphorylation of p38 MAPK and JNK, but not ERK1/2 in the lumbar spinal cord of EAE mice. The expressions of p38 MAPK, JNK, ERK1/2 and their phosphorylation expression were analyzed by Western blot. Statistically significant data are indicated by asterisks (^**^P < 0.01 and ^****^P < 0.0001). ns, not significant.

T cell activation depends upon phosphorylation of MAPKs, which plays a critical role in the regulation of immune responses. MAPKs signaling, including p38 MAPK, ERK1/2, and JNKs, plays important roles in Th17 cell differentiation, which is a central player in MS and EAE (28, 29). We evaluated the expressions of MAPK signaling in the lumbar spinal cord of different groups by Western blot. We found that the level of phosphorylation of p38 MAPK, ERK1/2 and JNK was significantly increased in the lumbar spinal cord of EAE mice. Both *C. butyricum* and norfloxacin treatment suppressed the elevation of p38 MAPK and JNK, whereas the phosphorylation of ERK1/2 was not affected in the EAE mice (Figure 6).

## Discussion

Gut microbiota, particularly bacterial community has been proposed as an important environmental factor which can intervene and modulate CNS autoimmunity. Here, we demonstrated that altering gut flora by *C. butyricum* and norfloxacin could alleviate EAE in C57 BL/6 mice. In this experiment, the timing and duration of the two interventions were different. *C. butyricum* (5.0 × 106 CFU/mL) treatment started at 3–4 weeks aged mice because weaning period in C57 BL/6 mice is important for the development of its immune system (7, 16, 30). Early life antibiotics intervention can predispose an individual to develop CNS autoimmunity diseases in latter life, thus norfloxacin (5 mg/kg intragastrically/daily) treatment started 1 week prior to EAE induction (31, 32). We found that both interventions could ameliorate EAE while the alteration of gut microbiota composition is different. *C. butyricum* treatment increased the abundance and diversity of the gut microbiota in EAE mice. The results are consisted with previous reports that *C. butyricum* could regulate gut homeostasis and improve gastrointestinal function as probiotics. Also, it reconfirmed that weaning period of age is an ideal time for probiotic intervention (7, 16, 30). Norfloxacin treatment significantly reduced both the abundance and diversity of the gut microbiota in EAE mice. The reduced abundance and diversity of gut microbiota in different situation could produce discrepancy results after norfloxacin treatment (31, 32). It was reported that norfloxacin administration for 2 weeks could suppress gut flora thus enhance glucose tolerance in diabetes mice and show beneficial effects for improving glycemic control (31), while norfloxacin treatment for the same time duration induced gut drug resistance gene expression and restricted the growth of collembolans (32). In our experiment, 5 mg/kg/d norfloxacin treatment for 1 week before EAE immunization produced beneficial effects on EAE mice. The results remind us that manipulating gut microbiota interventions on EAE should consider the timing, dosage and duration, which may consequently lead to divergent outcomes.

In the phylum level analysis, supply of *C. butyricum* and norfloxacin both can significantly increase the abundance of *Bacteroidetes* and decrease the richness of *firmicutes* in EAE mice. *Firmicutes* and *Bacteroidetes* are the two major members of gut bacteria at the phylum level and play an important role in modulating host inflammation and immune status (33). And the elevated *firmicutes/bacteroidetes* ratio (F/B ratio) can describe pro-inflammatory environment and immunological imbalance characteristic of autoimmune disorders (34, 35). It was reported that *bacteroidetes* can regulate intestinal epithelium function and reduce inflammation (34, 35). Some of them produce short chain fatty acids (SCFAs) including acetate, propionate and butyrate. SCFAs were reported to promote production of anti-inflammatory cytokines transforming growth factor-β (TGFβ) and IL-10, then activate Treg cells and modulate inflammatory and immune responses in host (36). *Firmicutes* are thought to have important and core roles in the host's metabolism (37). It could lead to the increased production of metabolic endotoxins like lipopolysaccharides, which was able to enter the blood streams and caused the chronic inflammation (38). We found that decreased F/B ratio in *C. butyricum* and norfloxacin treated EAE mice. This may be one of the important factors that contribute to the alleviation of EAE. Moreover, norfloxacin treatment increased abundance of *Verrucomicrobia* which is usually used as probiotics for its capacity of reducing inflammatory immune responses (25).

When it comes to the genus level analysis, we found that treatment with *C. butyricum* was associated with increased relative abundance of *Prevotella*, while genus *Akkermansia* and *Allobaculum* increased in norfloxacin treated EAE mice. In MS patients, *Prevotella* was decreased without treatment but increased after treated with disease-modifying drugs such as interferon-β (IFN-β) and glatiramer acetate (GA) (3). *Prevotella* could also metabolize phytoestrogens into beneficial metabolites and promote the generation of butyrate (one of SCFAs) which has anti-inflammatory and immune-modulating effects (39–41). *Akkermansia* and *Allobaculum* are mucin-degrading bacteria and increased in norfloxacin treated EAE mice. Whether the increase of *Akkermansia* could exhibit beneficial activities by mucin degradation is controversial (42–47). It was reported that *Akkermansia* have immunoregulatory effects on converting mucin to SCFAs, and attenuating inflammation in adipose tissue through induction of Foxp3 regulatory T cells, and suppression of IL-6 and IL-1β (42, 43). And *Akkermansia* also plays a reverse role in degrading the mucus layer in proinflammation function (44, 45). The discrepancy suggests that whether *Akkermansia* produce pro- or anti-inflammatory effects depending on the immune status of the host. *Allobaculum* was reported to be inversely correlated with dietary-induced inflammation markers, including leptin and IL-22 (46, 47). Furthermore, both *C. butyricum* and norfloxacin treatment reduced *Desulfovibroneceae* and *Ruminococcus* species which are reported to be the IL-17 provocating bacteria (48, 49).

Moreover, we found although both *C. butyricum* and norfloxacin could decrease F/B ratio and alleviate EAE, the genus level analysis of gut microbiota was obviously different. This suggested that the overall change and outcome of intervention of gut microbiota is intricate. Each strain of bacteria may have good or bad or no effect on EAE, and the balance of the all the bacteria may determine the overall effect of gut microbiota. The final outcome of an intervention may depend on whether it could promote the good bacteria and/or inhibit bad bacteria to achieve a balanced and beneficial community in EAE mice. In different situation, a balanced community of gut microbiota may show inter-individual differences, so “good” or “bad” bacteria may be a shift status at different condition. And this can explain why the specific bacterial strains have distinct effects in different hosts as well as the same treatment may produce divergent outcomes in different status in autoimmune diseases.

Gut microbiota can modulate the immune response in a variety of ways, such as affecting antigen presentation and regulating the production of cytokines and the function of lymphocytes (50). We further investigated the effects of *C. butyricum* and norfloxacin treatment on T cell responses which play an important role in regulation and propagation of encephalitogenic immune damage (51). We found that *C. butyricum* treatment reduced CNS-, LN-, small intestine-, colon-, and spleen- infiltration of pro-inflammatory Th17 cells, and increased the percentages of Tregs in LN, colon, and small intestine. The above mentioned gut microbiota alternation may be responsible for this effect. As Th17 cells are considered as the main population of pathogenic T cells driving EAE while Treg cells are of paramount importance for suppressing inflammatory immune responses in EAE (25, 51, 52), the change of T cells response in our study account for the reduced clinical and neuropathology score in EAE mice in our experiment. The results are consistent with a series of researches in which beneficial effects of gut microbes intervention with probiotics on EAE were achieved through generation of Tregs (53–55). No significant differences were observed in the IFN-γ producing Th1 cells in CNS, LN, small intestine and colon. The results suggested that the Th1 response in these locations may not be affected by *C. butyricum* treatment in this experiment. Interestingly, *C. butyricum* decreased the frequency of Th1 cells in spleen. Since spleen is an important peripheral immune organ in EAE, reduced Th1 in spleen may attenuate the severity of EAE. Further studies to explore the relationships of *C. butyricum*, the gut microbes and the Th1 response are highly wanted.

Norfloxacin displayed reduced CNS-, LN-, colon-, and spleen- infiltration of pro-inflammatory Th17 cells and increased the percentages of Tregs were observed in LN, colon, and small intestine. Our results are consistent with previous studies which found that the protective effect of antibiotic treatment in EAE is related with a regulation of the abnormal in T cell responses including increasing secretion of Treg cells (10, 56). Besides the reduced F/B ratio and *Desulfovibroneceae* and *Ruminococcus* species, increased the *Akkermansia* and *Allobaculum* may also contribute to the beneficial effects. However, the Th17 of small intestine was not decreased in EAE mice. This result may be partly due to that Th17 cells mostly differentiate in small-intestinal lamina propria (57–59). And it may have better resistance capacity to norfloxacin treatment. No significant differences were noted in the percentages of Th1 cells in norfloxacin treatment. Our results verified the extra-anti-infective effects of norfloxacin on hosts through the modulation of gut microbiota and immune response in EAE mice. And this is consistent with previous reports that norfloxacin could enhance a regulatory T cell-mediated inflammatory control in cirrhosis by maintaining low IL-2 and IFN-γ levels and stimulating IL-10 production (13, 14).

The molecular mechanisms behind the protective effect of *C. butyricum* and norfloxacin may be associated with MAPK signaling. We found that the p38 MAPK and JNK, but not ERK1/2, were not activated by *C. butyricum* and norfloxacin administration. p38 MAPK-mediated signaling, a well-characterized integrator of environmental stressors (60), regulates numerous cellular events associated with the inflammatory response, cell proliferation and cell survival and induction of Th17 cell differentiation (60, 61). Additionally, the coupling of both the p38 and JNK-MAPK pathways to T cell receptor signaling might allow for lineage-specific signals in T cell differentiation (62). Collectively, these researches suggest that alterations in MAPK signaling may be associated with changes in the microbiota.

In conclusion, interventions with *C. butyricum* and norfloxacin influenced the composition of gut microbiota and, consequently, modulated the immune response in EAE mice. These findings support the idea that gut microbiota modulation has the potential to the future treatment of MS. The results also remind us that factors such as timing, duration and dosage must be carefully considered in probiotics and antibiotic-associated manipulation of the gut microbiota. The “good bacteria” are not always beneficial and “bad bacteria” are not always bad for hosts at any conditions. Recovery from dysbiosis by gut microbiota intervention on MS patients should consider the cross-talk between gut bacteria and immune status of the host. This study contributes to the pool of knowledge regarding the complex relationship between gut microbiota and CNS autoimmunity.

## Data Availability

The raw data supporting the conclusions of this manuscript will be made available by the authors, without undue reservation, to any qualified researcher.

## Ethics Statement

This study was carried out in accordance with the recommendations of guidelines for animal care, created by according to the National Institutes of Health Guide for Care and Use of Laboratory Animals and approved by the Bioethics Committee of South China Agricultural University (Approval ID: 2018-D006). The protocol was approved by the Bioethics Committee of South China Agricultural University.

## Author Contributions

XC designed the research and charge correspondence. HC, XiM, ZC, XL, LS, and LM performed the experiments. HC, YL, XiM, and XC wrote the manuscript. All the authors read and approved the final manuscript.

### Conflict of Interest Statement

The authors declare that the research was conducted in the absence of any commercial or financial relationships that could be construed as a potential conflict of interest.
